# Color first, space next, orientation last: A temporal comparison of retro-cue effects in visual working memory

**DOI:** 10.3758/s13421-025-01789-8

**Published:** 2025-09-17

**Authors:** Lijing Guo, Dan Nie, Penglan Liu, Lingcong Zhang, Chaoxiong Ye

**Affiliations:** 1 School of Education, Anyang Normal University, Anyang, China; 2 Department of Psychology, University of Jyvaskyla, Jyvaskyla, Finland; 3 Department of Educational Science and Technology, Minnan Normal University, Zhangzhou, China

**Keywords:** Visual working memory, Feature retro-cue, Spatial retro-cue, Retro-cue benefit

## Abstract

Retro-cues can enhance performance in visual working memory (VWM) tasks by directing internal attention to relevant items. While spatial retro-cues have been extensively studied, less is known about how different types of feature retro-cues (e.g., color, orientation) compare in effectiveness and temporal dynamics. Across four experiments, we directly contrasted spatial, color, and orientation retro-cues in dual-feature memory tasks and systematically varied cue–probe delays (50–650 ms) to track the time course of retro-cue benefits (RCBs). Results revealed a processing speed hierarchy: color retro-cues elicited larger benefit than spatial retro-cue at the shortest delays (50 ms), followed by spatial retro-cues (200 ms), whereas orientation retro-cues required longer delays (500 ms or more). Notably, color retro-cues produced stronger or more rapid RCBs than spatial cues, suggesting they engage attentional mechanisms more efficiently. In contrast, orientation retro-cues were ineffective unless participants were explicitly required to encode both features. These findings indicate that different retro-cue types differ not only in efficacy but also in how quickly they can modulate memory performance, reflecting feature-specific constraints in attentional selection and cue encoding. Our results challenge the assumption that all feature cues operate uniformly and highlight the importance of considering cue type, task goals, and retrieval context in models of selective attention within working memory.

## Introduction

Visual working memory (VWM) is a crucial cognitive system for short-term information storage and processing. However, its capacity is limited, typically able to maintain only three to four simple items at the same time (Awh & Vogel, 2025; Cowan, 2001; Luck & Vogel, 1997, 2013; Luria et al., 2016). These capacity constraints impose significant limitations on broader cognitive abilities. As a result, many studies have focused on uncovering the mechanisms underlying these limitations and exploring ways to enhance VWM performance. When working with larger memory sets, individuals can use internal attention mechanisms (refers to the selection, modulation, and maintenance of internally generated information) to selectively focus on certain representations within VWM, typically a single item, in order to reduce cognitive load and improve memory performance (Cowan, 1998; Oberauer, 2002). The role of internal attention in VWM has become a key area of research, with ongoing studies exploring how attention allocation influences the capacity of VWM and memory performance (Fougnie & Marois, 2006; Liang et al., 2019; Lu et al., 2017; Robison & Unsworth, 2017; Serin & Günseli, 2023; Ye et al., 2018, 2019, 2021; Zhang et al., 2018; Zheng et al., 2024).

Retro-cues are regarded as tools to orient internal attention to the cued item during VWM processing (Griffin & Nobre, 2003; Landman et al., 2003; Souza & Oberauer, 2016). In a typical retro-cue experiment, after the memory array offset, an arrow displayed at the center of the screen points to the location of an item (spatial retro-cue) that will be the probe in a subsequent detection task. A validly retro-cued item can be accessed faster and more accurately compared with conditions where no cue or an uninformative cue was presented (Fu et al., 2022; Guo et al., 2024; Janczyk & Berryhill, 2014; Liu et al., 2023, 2024a, b, 2025; Makovski & Jiang, 2008; Matsukura et al., 2007; Rerko et al., 2014; Souza & Oberauer, 2016; Ye et al., 2016). This phenomenon is referred to as the retro-cue benefit (RCB).

Empirical research has demonstrated that time is required after retro-cue presentation for cued items to achieve enhanced memory performance. For instance, Souza, Rerko, Lin et al. (2014a) compared retro-cue conditions with different postcue delays with a no-cue condition involving an early probe. In the no-cue condition with an early probe, the probe array can be regarded as functionally equivalent to a retro-cue in the valid-cue condition, as both the retro-cue and the probe array were presented after the same fixed interval following the memory array. Memory performance in the no-cue condition was significantly worse than in the retro-cue condition, and RCBs increased with longer postcue time. These findings suggest that RCBs rely on a temporal process of attentional reallocation and memory enhancement, with adjustments in VWM resource allocation taking time to develop. Consequently, this suggests that RCBs are greater when the probe appears after a delay following the retro-cue rather than immediately after retro-cue onset.

To fully understand the mechanisms underlying RCBs, it is important to distinguish between two primary types of retro-cues: spatial retro-cues and feature retro-cues. Spatial retro-cues are centrally presented cues that indicate the spatial location of the to-be-probed item. In contrast, feature retro-cues are centrally presented cues that indicate a non-probed feature dimension of the target item in a multifeature memory task. For example, participants are asked to remember both the color and orientation of a set of items, and then they receive a red color retro-cue, directing their attention to the orientation of the memory item that contains the red color feature (Pertzov et al., 2013). In addition to color, other features such as orientation and shape have also been used as feature retro-cues (Arnicane & Souza, 2021). Although significant differences exist between spatial and feature retro-cues, previous studies have demonstrated that both types of retro-cues can effectively produce RCBs (Arnicane & Souza, 2021; Li & Saiki, 2015; Pertzov et al., 2013).

Notwithstanding, the mechanism by which participants use spatial retro-cues to enhance VWM performance may be entirely different from the processing mechanism involved in using feature retro-cues to achieve a comparable level of enhancement (Carrasco, 2011). Feature retro-cues directly highlight a target’s specific feature (e.g., color or orientation) of a multi-feature item, facilitating a goal-directed selection mechanism without relying on spatial information. This direct selection mechanism is closely tied to sensory-driven processing (Andersen et al., 2009; Heuer & Schubö, 2016; Heuer et al., 2016). In contrast, spatial retro-cues operate by indirectly guiding attention to the target’s location using visual indicators, like arrows. These cues rely on spatial coordinates to enhance the representation of the target’s location, enabling efficient filtering of irrelevant information in a visually complex field. The attentional mechanisms engaged by spatial retro-cues are rooted in positional enhancement and spatial filtering (Duarte et al., 2013; Schneider et al., 2016; Wang & van Ede, 2025). These differences suggest that the temporal dynamics of RCB generation by feature retro-cues might differ from those by spatial retro-cues.

Previous research has conducted initial explorations into the temporal dynamics of RCBs, but only focused on spatial retro-cues. These studies have consistently demonstrated that a certain amount of time is required for spatial RCBs to emerge. Researchers have investigated this temporal aspect by manipulating the cue–probe delay, defined as the interval between the onset of the spatial retro-cue and the probe display. For instance, Tanoue and Berryhill (2012) examined cue–probe delays ranging from 100 to 700 ms in a VWM task. Their results revealed that significant RCBs emerged after 300 ms, with no Further increase in magnitude at longer delays, which indicates that the generation of spatial RCBs requires at least 300 ms. Besides, the studies by Souza et al. (2016) and van Moorselaar et al. (2015) also suggest that individuals require 300–500 ms or longer to effectively use spatial retro-cues for memory enhancement. While we have a basic understanding of the time required for using spatial retro-cues, there is scant literature on the time-course of feature retro-cue use. To the best of our knowledge, only two studies varied cue–probe delay after a feature retro-cue. In two experiments (one using spatial retro-cues, the other using retro-color cues), Pertzov et al. (2013) tested cue–probe delays of 300, 1,000, and 3,000 ms for both spatial and color retro-cues. They observed no benefit at 300 ms for either cue type, but significant benefits emerged at 1,000 ms and increased Further at 3,000 ms, which was likely due to a decline in performance in the no-cue condition over time. One experiment by Arnicane and Souza (2021) manipulated cue–probe delays of 500, 1,000, and 1,500 ms using a shape retro-cue, and found that all intervals led to significant RCBs. However, these studies did not investigate in fine detail the minimum time required for feature retro-cues to produce a benefit, nor did they directly compare this time course with that of spatial retro-cues. As a result, a critical gap remains in our understanding of the temporal dynamics of RCBs associated with different cue types.

Our study aims to explore the differences in the temporal dynamics of spatial and feature retro-cues in enhancing VWM performance. By systematically manipulating cue–probe delays under different cue type conditions, we seek to identify differences in the time required for each cue type to effectively improve memory performance. Building upon the difference between spatial and feature retro-cues, we formulated hypotheses about their temporal dynamics. These retro-cues operate through distinct mechanisms, leading to two opposing predictions.

The first hypothesis states that feature retro-cues directly highlight a specific feature of a memory item, whereas spatial retro-cues rely on positional information to redirect attention to the target indirectly. This fundamental difference in cueing approaches allows for the hypothesis that feature retro-cues may be more efficient, requiring less time to produce RCBs as they avoid the intermediary attentional conversion process inherent to spatial retro-cues. Additionally, feature retro-cues (e.g., color cue) directly present a feature from the memory item without introducing extraneous information, but spatial retro-cues introduces extraneous information (e.g., the arrow cue), and participants may need some time to process this additional information. Therefore, feature retro-cues may produce RCBs more quickly than spatial retro-cues due to their direct and interference-free cueing mechanism.

The second hypothesis is based on studies of feature-based attention. For example, feature-based attention is known to spread globally (Liu & Hou, 2011; Liu & Mance, 2011; Saenz et al., 2003), which takes time (Stoppel et al., 2012). Furthermore, in a study comparing spatial and feature precues at different cue-to-target intervals, it was found that spatial cues enhanced perceptual performance at a shorter temporal delay than feature (motion direction) cues (Liu et al., 2007). These results suggest that spatial cues engage a more direct process than feature cues, hence act faster. More specifically in VWM research, Heuer and Schubö (2016) demonstrated that feature retro-cues facilitate flexible attention allocation across the entire VWM layout, free from the constraints of physical spatial arrangements. In contrast, spatial retro-cues are inherently limited by the physical configuration of the memory array. This flexibility suggests that feature retro-cues may engage more complex and resource-intensive cognitive processes, potentially requiring longer time delays to produce RCBs. In this case, generating feature RCBs may require longer time due to their involvement in more complex information processing.

Importantly, the diversity of feature retro-cues may also contribute to the observed differences in their temporal dynamics. Feature retro-cues can be further categorized based on the specific feature use, such as color retro-cues or orientation retro-cues. Each type of feature retro-cue may engage distinct cognitive processes due to the inherent properties of the feature itself. For instance, color is a highly salient and stimulus-driven feature that can be processed in parallel, allowing for rapid attentional allocation (Andersen et al., 2009; Carrasco, 2011). In contrast, orientation may require more sequential processing, as participants need to compare the cued feature against all memory items individually (Heuer & Schubö, 2016). This variation in processing demands could lead to differences in the time required to produce RCBs for different feature retro-cues. Therefore, to fully understand the distinction between spatial and feature retro-cues, it is essential to consider the heterogeneity of feature retro-cues and their unique cognitive processing mechanisms.

Investigating these hypotheses will not only enhance our understanding of the differences in the temporal dynamics involved in using spatial and feature retro-cues to improve VWM performance but also provide deeper insights into the mechanisms underlying RCBs across different cue types. By contrasting the temporal dynamics of spatial and feature retro-cues, the research can reveal how different cognitive and neural processes contribute to attentional modulation of VWM content and how such modulations improve retrieval from this system.

## Experiment 1

In Experiment 1, participants performed a retro-cue recall task in which they were required to memorize four dual-feature items: color and orientation. To ensure stable consolidation of memory representations in VWM, retro-cues were presented 500 ms after memory array offset, we provided either a spatial retro-cue, a color retro-cue, or no cue. Consistent with the studies of Li and Saiki (2015), Pertzov et al. (2013), and Heuer and Schubö (2016), we used a color cue as the feature retro-cue. Participants were required to use the color feature as a retro-cue to redirect their attention to the item matching the cued feature. Following the cue presentation, we systematically varied the cue–probe delays to assess the temporal dynamics of RCBs. While previous studies have indicated that a delay of 300–500 ms is sufficient to produce spatial RCBs (Arnicane & Souza, 2021; Pertzov et al., 2013; Souza, Rerko, Oberauer, 2014b; Souza & Oberauer, 2016; Souza et al., 2016; Tanoue & Berryhill, 2012; van Moorselaar et al., 2015). It is, therefore, still uncertain whether color retro-cues require less or more time compared with spatial retro-cues. So, in this experiment, we included delays both below 300 ms and above 500 ms, resulting in five delays: 50 ms, 200 ms, 350 ms, 500 ms, and 650 ms. The primary goal of this experiment was to explore and compare the time required for RCBs to emerge under spatial and color retro-cues.

### Method

#### Participants

Based on the previous study by Pertzov et al. (2013), we anticipated a similar effect size (η_p_^2^ = 0.241) for our experimental design. A power analysis conducted using G*Power (Version 3.1.9.2; Faul et al., 2007) suggested that eight participants were required to achieve 95% power at an alpha level of 0.05. For Experiment 1, we recruited 18 participants (aged 18–22 Years, mean age = 19.611 ± 0.257 Years; 16 women, two men), exceeding the sample size (*n* = 17) used by Rerko et al. (2014) in their retro-cue study. Participants were undergraduate or postgraduate students with normal or corrected-to-normal vision and no history of neurological issues. For all studies reported here, participants provided written informed consent and received monetary compensation. The protocol of each reported study was approved by the Ethical Committee of Minnan Normal University and adhered to the Declaration of Helsinki (2008).

#### Stimuli and materials

The memory stimuli were the same as the corresponding stimuli in our previous study (Ye et al., 2016, 2021). The memory array consisted of four colored bars (1.1° in length, 0.4° in height), each with a specific orientation, presented on a gray background. The color and orientation of each memory stimulus were randomly selected from 360 possible colors (1–360, in 1° steps) and 180 possible angles (1–180, in 1° degrees). A palette of 360 colors was used as the same as we used in our previous studies (Ye et al., 2016, 2021). The bars could be presented in four possible locations, located at the corners of an invisible square (eccentricity, 3°), with any two bars separated by at least 30 orientation degrees and 60 color steps. In Experiment 1, participants were required to recall the orientation of the probed item. Three types of trials were used: spatial retro-cue, color retro-cue, and no cue. In spatial retro-cue trials, two black arrows appeared in the center of the screen pointing to the location of the probed item. The color retro-cue was a square filled with the color of the to-be-probed item. For example, if an item in the memory array was red with a orientation of 75°, its color cue would display a red square in the center of the screen. No cue refers to a condition where nothing was displayed at the screen, with no information provided to the participants indicating which item will be probed. The probe display consisted of a vertical white bar (1.1° × 0.4°, presented at fixation) and a white square (1.2° × 1.2°) marking the location of the probed item. The stimuli were presented on a 19 LCD monitor (1,280 × 1,024 pixel), and participants viewed the display at a distance of 60 cm in a dark room.

#### Procedure

**Fig. 1:**
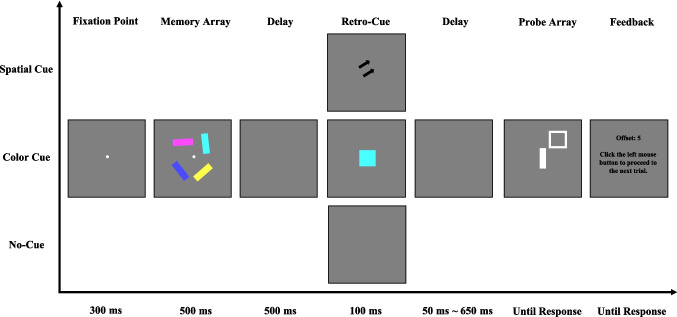
Illustration of the trial structure in Experiment 1. In the spatial retro-cue condition, the retro-cue consisted of two arrows pointing to the location of the probed item. In the color retro-cue condition, the retro-cue was a square filled with the color of the cued item. In the no-cue condition, no retro-cue was presented. (Color figure online)

Each trial (see Fig. 1) began with a 300-ms central fixation cross, followed by a 500-ms presentation of the memory array. Participants were instructed to remember both the spatial location and the visual features (color and orientation) of each item. After a 500-ms delay, a retro-cue (spatial or color) or no-cue was presented for 100 ms. In the spatial retro-cue condition, a spatial cue was shown. In the color retro-cue condition, a color cue was presented. All retro-cues were 100% valid. In the no-cue condition, the screen remained blank for 100 ms, providing no information about the to-be-probed item. After a variable cue–probe delay (50 ms, 200 ms, 350 ms, 500 ms, or 650 ms), the probe display appeared. In the no-cue condition, since no cue was presented, these cue–probe delays effectively corresponded to different memory–probe delays (i.e., 650 ms, 800 ms, 950 ms, 1,100 ms, or 1,250 ms, respectively). Participants used the mouse to rotate the central white bar to match their memory of the target’s orientation, which was indicated by the white square at its original location. Once satisfied, participants confirmed their response by clicking the left mouse button. Following this, feedback was provided, displaying the absolute angular error between the reported and actual orientations, with smaller values indicating better performance. The experiment consisted of 420 trials, with each participant completing 28 trials in each of the 15 experimental conditions (3 cue types: spatial retro-cue vs. color retro-cue vs. no cue × 5 cue–probe delays: 50 ms vs. 200 ms vs. 350 ms vs. 500 ms vs. 650 ms). Participants completed 20 practice trials before the main experiment to familiarize themselves with the task.

#### Data analysis

Data were analyzed using JASP (Version 0.19.0.0) to address two primary research questions:

1. Whether memory performance improved over time within each cue condition, and
2. Whether spatial and color retro-cues produced RCBs, and how quickly these benefits emerged.

Participants’ VWM performance was measured by calculating the absolute angular deviation between the reported orientation and the actual orientation of the probed item, referred to as the “offset.” Smaller offsets indicated better memory performance. To examine the interaction between cue type and cue–probe delay, we conducted a 3 (cue type: spatial retro-cue, color retro-cue, no cue) × 5 (cue–probe delay: 50 ms, 200 ms, 350 ms, 500 ms, 650 ms) repeated-measures analysis of variance (ANOVA). To assess temporal changes in performance within each cue condition, we conducted planned two-tailed paired-samples *t* tests comparing offset values between adjacent delay intervals (i.e., 50 vs. 200 ms, 200 vs. 350 ms, 350 vs. 500 ms, and 500 vs. 650 ms) for each cue condition separately. To quantify the magnitude of retro-cue benefits, we computed an RCB index for each participant using the following formula:

1 $$\text{RCB index}= \frac{{\mathrm{Offset}}_{\text{No cue}}-{\mathrm{Offset}}_{\mathrm{Retro}-\mathrm{cue}} }{{\mathrm{Offset}}_{\text{No cue}}}$$

where ${\mathrm{Offset}}_{\text{No cue}}$ refers to the mean offset in the no-cue condition, and ${\mathrm{Offset}}_{\mathrm{Retro}-\mathrm{cue}}$ refers to the mean offset in a given retro-cue condition. This allowed us to calculate separate RCB indices for the spatial and color retro-cue conditions. Positive values indicate a performance benefit from the cue, whereas negative values reflect a cue-induced cost. Larger RCB indices reflect greater cue-induced memory enhancement. For each cue–probe delay, separate two-tailed one-sample *t* tests were conducted to compare the RCB index against zero for both cue types, determining whether the cues provided a statistically reliable benefit relative to the no-cue condition. We also directly compared the RCB indices of spatial and feature (color) retro-cues using two-tailed paired-samples *t* tests. Effect sizes for ANOVAs were reported using partial eta squared (η_p_^2^), while Cohen’s *d* was used to index the effect size of significant *t*-test results. A significance level of *p* < 0.05 was applied for all statistical tests. Bayesian analyses were conducted to assess the strength of evidence for the observed effects. For the paired-samples and one-sample *t* tests, Bayes factors (BF_10_) were reported to quantify the evidence for the alternative hypothesis over the null hypothesis. For the repeated-measures ANOVA, we reported Bayes inclusion factors (BF_incl_) computed across all models, which estimate the overall evidence for including each main effect or interaction term in the model compared with models where that factor is excluded. The interpretation of Bayes factors followed conventional guidelines (Rouder et al., 2009; Wagenmakers et al., 2018): BF_10_ (or BF_incl_) > 3 was considered substantial evidence for the alternative hypothesis (or inclusion), whereas values < 1/3 were considered substantial evidence for the null hypothesis.

### Results

**Fig. 2:**
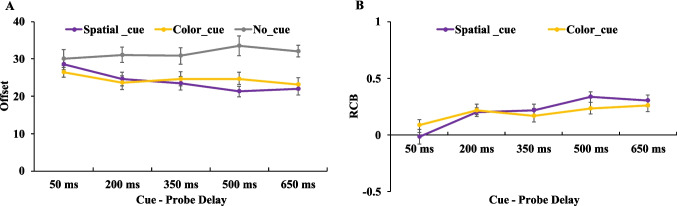
Illustration of the result of Experiment 1. Offset (**A**) and RCB index (**B**) results across different cue–probe delays for different cue type conditions. Error bars represent the standard errors of the mean. (Color figure online)

As seen in Fig. 2, a 3 (cue type: spatial retro-cue, color retro-cue, no cue) × 5 (cue–probe delay: 50 ms, 200 ms, 350 ms, 500 ms, 650 ms) two-way ANOVA on offset values revealed a significant main effect of cue type, *F*(2,34) = 17.357, *p* <.001, η_p_^2^ = 0.505, BF_incl_ > 1,000, a significant main effect of cue–probe delays, *F*(4,68) = 3.142,* p* =.020, η_p_^2^ = 0.156, BF_incl_ > 1,000, and a significant interaction between cue type and cue–probe delays, *F*(8,136) = 4.942, *p* <.001, η_p_^2^ = 0.225, BF_incl_ > 1,000.

To further explore the time course of memory performance in each cue type condition, a series of planned paired-samples *t* tests were conducted comparing adjacent cue–probe delays. The statistical results are summarized in Table 1. Between 50-ms and 200-ms delays, significant reductions in offset values were observed in both the spatial and color retro-cue conditions, indicating improved memory performance. However, no significant changes were observed at longer delays (i.e., between 200 and 350 ms, 350 and 500 ms, or 500 and 650 ms). These findings suggest that retro-cue benefits emerged rapidly within the first 200 ms following cue offset and subsequently reached a performance plateau, with no further improvement from prolonging the cue–probe interval up to 650 ms. In contrast, the no-cue condition showed no significant changes in performance across the different cue–probe delays (Fig. 2A), indicating that the observed time-dependent improvement was specific to retro-cue conditions.

**Table 1:** *T *tests assessing changes in performance across cue type conditions as a function of cue–probe delay in Experiment 1

Delay	Condition	*t*(17)	*p*	*d*	BF_10_
**50 - 200 ms**	**Spatial**	2.767	.013	0.652	4.207
	**Color**	2.544	.021	0.600	2.879
	**No**	0.947	.357	0.223	0.360
**200 - 350 ms**	**Spatial**	0.872	.396	0.205	0.340
	**Color**	0.817	.425	0.193	0.326
	**No**	0.179	.860	0.042	0.247
**350 - 500 ms**	**Spatial**	1.686	.110	0.397	0.793
	**Color**	0.101	.921	0.024	0.244
	**No**	1.694	.108	0.399	0.802
**500 - 650 ms**	**Spatial**	0.600	.557	0.141	0.285
	**Color**	1.005	.329	0.237	0.378
	**No**	0.813	.427	0.192	0.325

*Note.* For each cue–probe adjacent delay interval, the first row reports the comparison for the offset of the spatial retro-cue condition, the second row reports the comparison for the offset of the color retro-cue condition, and the third row reports the comparison for the offset of the no-cue condition

To assess the presence and time course of RCBs, we computed an RCB index for each retro-cue condition. One-sample *t* tests were used to compare each RCB index against zero, and paired-sample *t* tests were conducted to compare the spatial and color RCB indices at each cue–probe delay (see Table 2). At the 50-ms delay, neither spatial nor color retro-cues produced statistically significant benefits relative to the no-cue condition. However, from 200 ms onwards, both cue types yielded robust and statistically significant RCBs (see Fig. 2B), suggesting that a minimum cue–probe delay of 200 ms is required for the retro-cue to reliably enhance memory performance. When directly comparing spatial and color RCB indices, we found a significant difference only at the 50-ms delay, where the color retro-cue produced a larger benefit than the spatial retro-cue. At longer delays (200–650 ms), no significant differences were observed between the two cue types, suggesting that once RCBs have emerged and reached their peak magnitude, further extending the cue–probe delay does not differentially affect the benefit conferred by spatial versus color retro-cues.

**Table 2:** *T *tests assessing RCB indices for spatial and color retro-cues across cue–probe delays in Experiment 1

Delay	Condition	*t*(17)	*p*	*d*	BF_10_
**50 ms**	**Spatial**	0.256	.801	0.060	0.250
	**Color**	1.900	.075	0.448	1.061
	**Spatial vs. Color**	2.298	.035	0.542	1.931
**200 ms**	**Spatial**	5.476	< .001	1.291	623.816
	**Color**	4.182	< .001	0.986	56.176
	**Spatial vs. Color**	0.396	.697	0.093	0.261
**350 ms**	**Spatial**	3.937	.001	0.928	35.444
	**Color**	2.807	.012	0.662	4.504
	**Spatial vs. Color**	0.926	.368	0.218	0.354
**500 ms**	**Spatial**	7.047	< .001	1.661	> 1000
	**Color**	4.240	< .001	0.999	62.666
	**Spatial vs. Color**	2.031	.058	0.479	1.283
**650 ms**	**Spatial**	5.850	< .001	1.379	> 1000
	**Color**	4.777	< .001	1.126	171.581
	**Spatial vs. Color**	1.086	.293	0.256	0.406

*Note.* For each cue–probe delay, the first row reports the comparison between spatial RCB index and zero, the second row reports the comparison between color RCB index and zero, and the third row reports the direct comparison between spatial and color RCB indices.

### Discussion

In Experiment 1, we found that in the no-cue condition, participants’ memory performance remained stable across cue–probe delays, showing no significant fluctuations. In contrast, both spatial and color retro-cues led to improved memory performance with increasing cue–probe delays from 50 to 200 ms, with stable RCBs emerging from 200 ms onward. These findings suggest that retro-cues facilitate memory performance over time, particularly when sufficient processing time is available following the retro-cue (Pertzov et al., 2013). Our results indicate that both spatial and color retro-cues required approximately 200 ms (i.e., 300 ms after retro-cue onset) to produce stable RCBs. Once established, however, the magnitude of the RCBs did not further increase with longer delays.

Notably, we also found that at the 50-ms delay, the color RCB index was significantly larger than the spatial RCB index. This suggests that even at very short delays, participants were able to derive a greater performance benefit from color retro-cues than from spatial ones. However, as the cue–probe delay increased, the advantage provided by both cue types converged, yielding comparable levels of performance enhancement.

Thus, while we did not find strong evidence that one retro-cue type (spatial vs. color) led to an earlier onset of RCBs in a broad sense, our findings do suggest that color retro-cues may be used more rapidly and effectively than spatial cues under time-constrained conditions. This may reflect an inherent difference in the ease with which different retro-cue types can be processed and applied.

It is important to note that the memory items in our study were defined by both color and orientation features. Color, as a highly salient and stimulus-driven feature, tends to automatically capture attention (Hulleman, 2020). This intrinsic salience likely allows participants to isolate color features efficiently, without the need for extensive comparisons across memory items. As a result, color retro-cues may be rapidly and effectively used. In contrast, more abstract or less salient features, such as orientation, may require more effortful processing to generate retro-cue benefits. Therefore, the similarity in the time course of RCBs between spatial and color retro-cues observed in Experiment 1 may reflect the simplicity and efficiency of color processing specifically, and should not be generalized to all types of feature retro-cues without further empirical testing.

To further investigate whether the time course of RCBs differs between spatial and feature retro-cues, Experiment 2 was designed to directly compare spatial retro-cues with orientation retro-cues. Using orientation as the cued feature offers an ideal contrast for this purpose. First, unlike color, identifying a cued orientation may involve comparative processing across multiple memory items, potentially engaging more complex mechanisms such as top-down attentional modulation and fine-tuning of neural representations (Hao et al., 2018; Miller et al., 2014). Second, Experiment 1 used memory items defined by both color and orientation features, with color used as the retro-cue. By using orientation retro-cues in Experiment 2 while keeping the memory stimuli identical as Experiment 1, we were able to minimize variability during the encoding phase and only manipulate the differences in cue processing between spatial and feature retro-cues.

## Experiment 2

In Experiment 2, we replaced the color retro-cue with an orientation retro-cue while keeping the spatial retro-cue and no-cue conditions identical to those in Experiment 1. Participants were still required to memorize four dual-feature items (color and orientation). However, unlike in Experiment 1 where participants reported the orientation of the probed item, the task in Experiment 2 required participants to report the color of the probed item following the onset of probe display.

### Method

#### Participants

Twenty-one participants (ages 17–22 Years, mean age = 19.762 ± 0.308 Years; 18 women, three men) took part in the experiment. All participants were undergraduate or postgraduate students with normal or corrected-to-normal vision and no reported history of neurological or psychiatric disorders.

#### Stimuli and materials

The memory stimuli and spatial retro-cue used in Experiment 2 were identical to those in Experiment 1. In the orientation retro-cue condition, a new type of retro-cue was introduced: A white oriented bar was presented at the center of the screen, with its orientation matching exactly that of one of the items in the memory array. For example, if the probed item had a red color and an orientation of 75°, the orientation cue would be a white bar oriented at 75°. At the time of the probe display, participants were instructed to report the color of the cued memory item. The probe display consisted of a continuous color wheel (5.8° inner radius; 2.2° ring thickness) containing 360 hues, along with a white square marking the location of the probed item.

#### Procedure

**Fig. 3:**
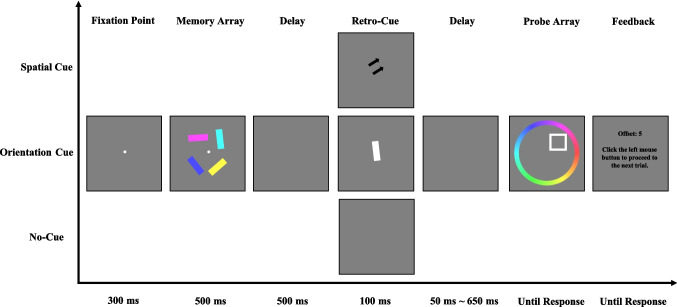
Illustration of the trial structure in Experiment 2. In the spatial retro-cue condition, the retro-cue consisted of two arrows pointing to the location of the probed item. In the orientation retro-cue condition, the retro-cue was a white bar with orientation of the cued item. In the no-cue condition, no retro-cue was presented. (Color figure online)

The procedure closely followed that of Experiment 1. Each trial began with a central fixation cross displayed for 300 ms, followed by a memory array of four colored bars shown for 500 ms. Participants were instructed to remember both the location and the feature information (color and orientation) of all items. After a 500-ms delay interval, a retro-cue (spatial or orientation) or no-cue was presented for 100 ms. In the spatial retro-cue condition, a spatial cue was shown. In the orientation retro-cue condition, an oriented white bar appeared at fixation. All retro-cues were 100% valid. In the no-cue condition, the screen remained blank for 100 ms with no informative cue. Following a variable delay (50 ms, 200 ms, 350 ms, 500 ms, or 650 ms), the probe display appeared. This display included a white square located at one of the item positions from the memory array, indicating the target location. The target item location was indicated by a white square. Participants were instructed to use the mouse to select the color from the color wheel that best matched the remembered color of the target item. After response confirmation, feedback was provided, displaying the absolute angular difference (in color degrees) between the selected and actual color value—smaller differences reflected better performance. The experiment consisted of 420 trials in total, fully crossed across three retro-cue conditions (spatial, orientation, and no cue) and five cue–probe delays (50 ms, 200 ms, 350 ms, 500 ms, and 650 ms), resulting in 28 trials per condition. Participants completed 20 practice trials before the main experiment to familiarize themselves with the task (see Fig. 3).

#### Data analysis

Participants’ recall performance was quantified by computing the absolute deviation between their reported color and the actual target color, referred to as “offsets,” which served as a measure of memory performance under different conditions. To examine the interaction between cue type and cue–probe delay, a 3 (cue type: spatial retro-cue, orientation retro-cue, no cue) × 5 (cue–probe delay: 50 ms, 200 ms, 350 ms, 500 ms, 650 ms) repeated-measures ANOVA on offset values was conducted. To assess temporal changes in performance within each cue type condition, a series of planned paired-samples *t* tests were conducted comparing offset values across adjacent cue–probe delays. Additionally, to evaluate the magnitude and time course of RCBs, we calculated an RCB index for each retro-cue (spatial or orientation) condition at each cue–probe delay. For each cue–probe delay interval, separate one-sample *t* tests were conducted for spatial and orientation retro-cues, testing whether the RCB index was significantly greater than zero—indicating a performance benefit relative to the no-cue condition. Furthermore, paired-samples *t* tests were conducted to directly compare the RCB indices between the spatial and orientation retro-cue conditions at each delay.

### Result

**Fig. 4:**
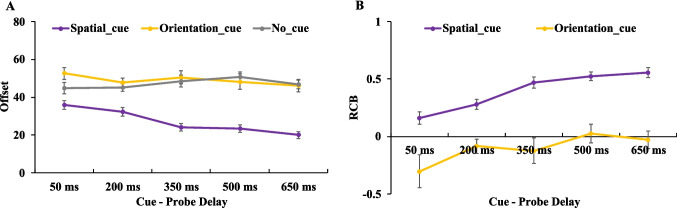
Illustration of the result of Experiment 2. Offset (**A**) and RCB index (**B**) results across different cue–probe delays for different cue type conditions. Error bars represent the standard errors of the mean. (Color figure online)

As shown in Fig. 4, a 3 (cue type: spatial retro-cue, orientation retro-cue, no cue) × 5 (cue–probe delay: 50 ms, 200 ms, 350 ms, 500 ms, 650 ms) two-way ANOVA on offset values revealed a significant main effect of cue type, *F*(2,40) = 82.588, *p* <.001, η_p_^2^ = 0.805, BF_incl_ > 1,000, and a significant main effect of cue–probe delays, *F*(4,80) = 4.618, *p* =.002, η_p_^2^ = 0.188, BF_incl_ > 1,000. as well as a significant interaction between cue type and cue–probe delays, *F*(8,160) = 4.205, *p* <.001, η_p_^2^ = 0.174, BF_incl_ > 1,000.

To further examine the time course of memory performance within each cue condition, planned paired-samples *t* tests were conducted comparing adjacent cue–probe delays (see Table 3). For the spatial retro-cue condition, no significant differences were observed between the 50 ms and 200 ms cue–probe delays, or between the 350-ms and 500-ms delays. However, significant performance improvements (i.e., decreased offset values) were found between 200 ms and 350 ms, and between 500 ms and 650 ms. In contrast, for both the orientation retro-cue and no-cue conditions, memory performance remained stable across all delay intervals, with no significant changes in offsets (Fig. 4A). These findings suggest that performance in the spatial retro-cue condition improved with increasing cue–probe delay, whereas performance in the orientation retro-cue condition and no-cue conditions was unaffected by the length of the delay.

**Table 3:** *T* tests assessing changes in performance across cue type conditions as a function of cue–probe delays in Experiment 2

Delay	Condition	*t*(20)	*p*	*d*	BF_10_
**50 – 200 ms**	**Spatial**	1.792	.088	0.391	0.882
	**Orientation**	1.553	.136	0.339	0.641
	**No**	0.170	.867	0.037	0.231
**200 – 350 ms**	**Spatial**	5.507	< .001	1.202	> 1000
	**Orientation**	0.882	.388	0.193	0.322
	**No**	1.036	.313	0.226	0.366
**350 – 500 ms**	**Spatial**	0.621	.542	0.135	0.271
	**Orientation**	0.564	.579	0.123	0.263
	**No**	0.611	.548	0.133	0.269
**500 – 650 ms**	**Spatial**	2.685	.014	0.586	3.763
	**Orientation**	0.504	.620	0.110	0.255
	**No**	1.299	.209	0.283	0.475

*Note.* For each adjacent delay interval, the first row reports the comparison for the offset of the spatial retro-cue condition, the second row reports the comparison for the offset of the orientation retro-cue condition, and the third row reports the comparison for the offset of the no-cue condition

To assess the presence and time course of RCBs, we computed the RCB index for each retro-cue condition and conducted one-sample *t* tests against zero at each cue–probe delay. We also directly compared the spatial and orientation RCB indices at each delay using paired-samples *t* tests (Table 4). In the spatial retro-cue condition, the RCB index was significantly greater than zero at all delay intervals from 50 ms to 650 ms, indicating a consistent and robust benefit regardless of the length of the cue–probe interval. In the orientation cue condition, however, the RCB index was significantly below zero at the 50-ms delay, suggesting a detrimental effect of the cue at short delays. At longer delays (200–650 ms), the RCB index did not differ significantly from zero at any time point, indicating that the orientation retro-cue neither benefited nor harmed performance beyond the shortest delay. Moreover, across all delay conditions, the spatial RCB index was significantly greater than the orientation RCB index, suggesting a clear advantage of spatial over orientation retro-cues in producing memory enhancement. These results indicate that spatial retro-cues begin to confer a performance benefit as early as 50 ms following cue presentation and maintain this benefit consistently over time. In contrast, orientation retro-cues initially disrupt performance at short delays and fail to provide any measurable advantage even at longer intervals.

**Table 4:** *T *tests assessing RCB indices for spatial and orientation retro-cues across cue–probe delays in Experiment 2

Delay	Condition	*t*(20)	*p*	*d*	BF_10_
**50 ms**	**Spatial**	2.958	.008	0.645	6.225
	**Orientation**	2.142	.045	0.467	1.491
	**Spatial vs. Orientation**	3.266	.004	0.713	11.252
**200 ms**	**Spatial**	6.166	< .001	1.346	>1000
	**Orientation**	1.370	.186	0.299	0.514
	**Spatial vs. Orientation**	7.672	< .001	1.674	>1000
**350 ms**	**Spatial**	9.765	< .001	2.131	> 1000
	**Orientation**	1.139	.268	0.249	0.403
	**Spatial vs. Orientation**	6.860	< .001	1.497	> 1000
**500 ms**	**Spatial**	13.437	< .001	2.932	> 1000
	**Orientation**	0.305	.763	0.067	0.237
	**Spatial vs. Orientation**	6.819	< .001	1.488	> 1000
**650 ms**	**Spatial**	12.546	< .001	2.738	> 1000
	**Orientation**	0.375	.712	0.082	0.242
	**Spatial vs. Orientation**	7.798	< .001	1.702	> 1000

*Note.* For each cue–probe delay, the first row reports the comparison between spatial RCB index and zero, the second row reports the comparison between orientation RCB index and zero, and the third row reports the direct comparison between spatial and orientation RCB indices

## Discussion

In Experiment 2, we found that participants’ memory performance in the no-cue condition remained stable across cue–probe delays, showing no significant improvement or decline. Similarly, in the orientation retro-cue condition, memory performance did not improve with increasing delays, and no reliable orientation retro-cue RCBs were observed at any delay interval. Notably, at the shortest cue–probe delay (50 ms) condition, the presence of an orientation retro-cue significantly impaired VWM performance. This impairment may be due to the orientation retro-cue itself acting as a form of visual interference: when the cue–probe interval is very short, participants may not have sufficient time to process the orientation retro-cue without disrupting ongoing memory maintenance or retrieval, particularly for the task-relevant feature (color). As a result, performance in the orientation retro-cue condition was worse than in the no-cue condition at the shortest cue–probe delay condition. In contrast, in the spatial retro-cue condition, memory performance improved as cue–probe delays increased, and significant spatial RCBs were evident from as early as 50 ms cue–probe delay. These findings suggest that spatial retro-cues effectively enhanced memory performance, whereas orientation retro-cues failed to produce measurable benefits under the current task demands.

The absence of orientation RCBs in Experiment 2 may be explained by two possibilities. One possibility is that participants were simply unable to use orientation retro-cues to produce RCBs, suggesting that the effectiveness of feature retro-cues may not generalize across different feature types. However, this explanation appears inconsistent with previous findings showing successful use of orientation retro-cues to enhance memory performance (Arnicane & Souza, 2021). A second possibility is that the task structure in Experiment 2 did not require participants to fully encode and maintain the non-probed feature (i.e., orientation). This may have created a strategic trade-off in memory resource allocation, where participants prioritized the probed feature (color) and ignored nonprobed feature (orientation), which was irrelevant for task performance. In such cases, participants may have encoded the items as colored squares rather than as integrated colored orientations. Consequently, the orientation retro-cue provided no helpful information—and potentially even served as a distractor—because it referred to a feature that had not been actively maintained. This strategic bias may have been particularly pronounced in Experiment 2, as color is a salient and easily encoded feature, whereas orientation is more abstract and cognitively demanding. Given the higher resource demands associated with encoding and maintaining orientation, it is plausible that participants did not successfully consolidate orientation information in VWM. If the orientation feature was poorly encoded or rapidly forgotten, the orientation cue would have no meaningful target representation to operate on—thus failing to produce a cueing benefit.

Thus, Experiment 3 was designed as a follow-up to Experiment 2, with a new task structure that explicitly required participants to encode and maintain both color and orientation features in VWM. By modifying the task so that successful performance depended on both color and orientation information, we aimed to ensure that orientation was encoded and maintained. If orientation RCBs emerge in Experiment 3, this would suggest that the absence of orientation RCBs in Experiment 2 was due to participants’ failure to maintain orientation representations—rather than any intrinsic limitation of orientation retro-cues themselves. This approach allows us to further clarify the boundary conditions under which feature retro-cues, and orientation retro-cues in particular, can enhance VWM performance.

## Experiment 3

In Experiment 3, we used a retro-cue recall task in which participants were required to memorize four items, each defined by both color and orientation. The design included three cue conditions: spatial retro-cue, orientation retro-cue, and no cue, with systematically varied cue–probe delays identical to those used in Experiments 1 and 2. The overall setup of Experiment 3 was nearly identical to that of Experiment 2, with one critical modification: the probe display no longer included a white square to indicate the location of the target item (as in Experiment 2). Instead, it included a white bar with a specific orientation, which served as a cue indicating which item’s color should be reported. Thus, the probe display in Experiment 3 consisted of a white oriented bar and a color wheel. As a result, to correctly report the color of the cued item, participants had to retrieve both the orientation and color of each memory item. This change ensured that participants were required to encode and maintain both features—color and orientation—during the memory maintenance interval. Compared with Experiment 2, this paradigm modification effectively forced the consolidation of both features into VWM, making it possible to assess the time course of orientation RCBs, if such benefits exist, under conditions where orientation information was reliably encoded and maintained.

### Method

#### Participants

Twenty participants (ages 18–22 Years, mean age = 19.750 ± 0.228 Years; 13 women, seven men) took part in the experiment. All participants were undergraduate or postgraduate students with normal or corrected-to-normal vision and no self-reported history of neurological disorders.

#### Stimuli and materials

The stimuli and materials were identical to those used in Experiment 2, with one exception: in Experiment 3, the probe display consisted of a color wheel containing 360 hues and a white oriented bar at the center, the orientation of which exactly matched one of the items from the memory array.

#### Procedure

**Fig. 5:**
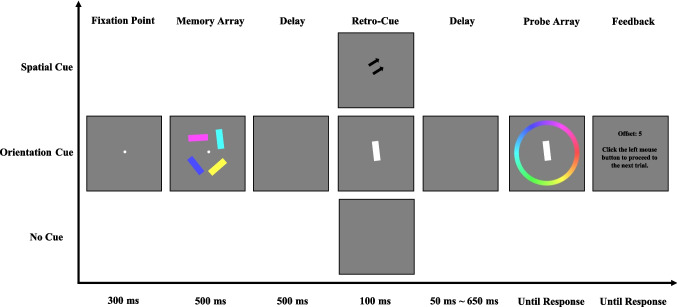
Illustration of the trial structure in Experiment 3. In the spatial retro-cue condition, the retro-cue consisted of two arrows pointing to the location of the probed item. In the orientation retro-cue condition, the retro-cue was a white bar with orientation of the cued item. In the no-cue condition, no retro-cue was presented. (Color figure online)

The procedure of Experiment 3 closely mirrored that of Experiment 2. Each trial began with a central fixation cross presented for 300 ms, followed by a memory array of four colored, oriented bars displayed for 500 ms. Participants were instructed to remember both the location and the feature information (color and orientation) of each item. After a 500-ms delay, a retro-cue (spatial cue, orientation cue, or no cue) was presented for 100 ms. All retro-cues were 100% valid. This was followed by a variable cue–probe delay (50 ms, 200 ms, 350 ms, 500 ms, or 650 ms), after which the probe display appeared. The probe display consisted of a white oriented bar displayed at the center, along with a color wheel surrounding it. The orientation of the white bar matched exactly one of the items from the preceding memory array. Participants were instructed to use the mouse to select from the color wheel the color that best matched the remembered color of the item that shared the same orientation as the probe bar. After confirming their response, participants received feedback indicating the absolute angular error (in color degrees) between the reported and actual color—smaller errors indicated better performance. The experiment comprised 420 trials in total, fully crossing three retro-cue conditions (spatial, orientation, and no cue) with five cue–probe delays (50 ms, 200 ms, 350 ms, 500 ms, and 650 ms), resulting in 28 trials per condition. Participants completed 20 practice trials before the main experiment to familiarize themselves with the task (see Fig. 5).

In Experiment 3, the white square location marker used in Experiment 2 was removed from the probe display. Instead, participants were required to use the orientation feature to identify the probed item. Crucially, this modification forced participants to encode and maintain both the color and orientation of each memory item. Without maintaining orientation information in VWM, participants would be unable to link the correct item and, consequently, could not accurately report its color. This design ensured that orientation information were essential for successful task performance and eliminated the possibility of alternative strategies that relied solely on color encoding.

#### Data analysis

Participants’ recall performance was quantified by computing the absolute deviation between their reported color and the actual target color, referred to as “offsets,” which served as a measure of memory performance under different conditions. To examine the interaction between cue type and cue–probe delay, a 3 (cue type: spatial retro-cue, orientation retro-cue, no cue) × 5 (cue–probe delay: 50 ms, 200 ms, 350 ms, 500 ms, 650 ms) repeated-measures ANOVA on offset values was conducted. Then, planned paired-samples *t* tests comparing offset values between adjacent delay intervals within each cue condition were conducted. We also compared the RCBs index for each cue condition at each cue–probe delay, as done in Experiment 2.

### Result

**Fig. 6:**
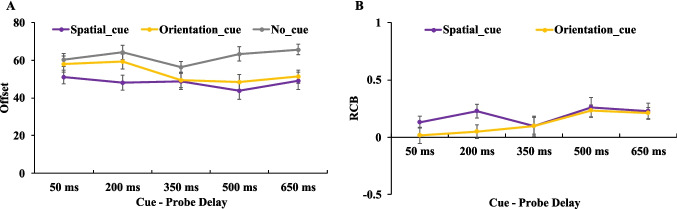
Illustration of the result of Experiment 3. Offset (**A**) and RCB index (**B**) results across different cue–probe delays for different cue type conditions. Error bars represent the standard errors of the mean. (Color figure online)

As seen in Fig. 6, a 3 (cue type: spatial retro-cue, orientation retro-cue, no cue) × 5 (cue–probe delays: 50 ms, 200 ms, 350 ms, 500 ms, 650 ms) two-way ANOVA on offset values revealed significant main effect for cue type, *F*(2,38) = 7.963,* p* =.001, η_p_^2^ = 0.295, BF_incl_ = 95.137, and cue–probe delays, *F*(4,76) = 3.487, *p* =.011, η_p_^2^ = 0.155, BF_incl_ = 5.621. Additionally, there was a significant interaction between cue type and cue–probe delays, *F*(8,152) = 2.717, *p* =.008, η_p_^2^ = 0.125, BF_incl_ = 14.537.

To further examine the time course of memory performance within each cue condition, planned paired-samples *t* tests were conducted comparing adjacent cue–probe delays. The statistical results are summarized in Table 5. From 200 ms to 350 ms, both the orientation retro-cue and no-cue conditions showed significant decrease in offset values. From 350 ms to 500 ms, the no-cue condition exhibited a significant increase in offset values, whereas the spatial retro-cue condition showed a significant decrease. For all other adjacent cue–probe delay comparisons, no significant changes in offset were observed (Fig. 6A). The results observed in the no-cue condition was characterized by a significant decrease in offset values from 200 ms to 350 ms, followed by a marked increase from 350 ms to 500 ms. This transient performance boost in the no-cue condition at 350 ms led to the observed fluctuation. In the spatial retro-cue condition, performance remained stable across delays from 50 ms to 350 ms, with a Further improvement between 350 ms and 500 ms. In the orientation retro-cue condition, performance was stable from 50 ms to 200 ms, but significantly improved between 200 ms and 350 ms.

**Table 5:** *T* tests assessing changes in performance across cue type conditions as a function of cue–probe delays in Experiment 3

Delay	Condition	*t*(19)	*p*	*d*	BF_10_
**50 - 200 ms**	**Spatial**	0.805	.431	0.180	0.310
	**Orientation**	0.550	.589	0.123	0.266
	**No**	1.084	.292	0.242	0.389
**200 - 350 ms**	**Spatial**	0.245	.809	0.055	0.239
	**Orientation**	3.589	.002	0.803	20.461
	**No**	2.690	.014	0.602	3.769
**350 - 500 ms**	**Spatial**	2.435	.025	0.545	2.420
	**Orientation**	0.316	.755	0.071	0.243
	**No**	2.638	.016	0.590	3.437
**500 - 650 ms**	**Spatial**	1.737	.099	0.388	0.827
	**Orientation**	1.055	.305	0.236	0.379
	**No**	0.985	.337	0.220	0.357

*Note.* For each adjacent delay interval, the first row reports the comparison for the offset of the spatial retro-cue condition, the second row reports the comparison for the offset of the orientation retro-cue condition, and the third row reports the comparison for the offset of the no-cue condition.

To assess the presence and time course of RCBs, we computed an RCB index for each retro-cue condition. One-sample *t* tests were used to compare each RCB index against zero, and paired-sample t-tests were conducted to compare the spatial and color RCB indices at each delay (see Table 6). For the spatial retro-cue condition, robust spatial RCBs were observed at all cue–probe delays except at 350 ms. In the orientation retro-cue condition, no significant orientation RCBs were observed at early delays (50–350 ms); however, significant orientation RCBs emerged at both the 500-ms and 650-ms delays (Fig. 6B). When directly comparing the spatial and orientation RCB indices, a significant difference was found only at the 200-ms delay, where the spatial RCB index was significantly greater than the orientation RCB index. At all other delays, the two cue types yielded statistically comparable RCB indices. These findings suggest that, by 200 ms after retro-cue offset, participants were already able to derive significantly greater memory benefits from spatial retro-cues compared with orientation retro-cues.

**Table 6:** *T* tests assessing RCB indices for spatial and orientation retro-cues across cue–probe delays in Experiment 3

Delay	Condition	*t*(19)	*p*	*d*	BF_10_
**50 ms**	**Spatial**	2.621	.017	0.586	3.332
	**Orientation**	0.250	.806	0.056	0.239
	**Spatial vs. Orientation**	1.537	.141	0.344	0.637
**200 ms**	**Spatial**	3.804	.001	0.851	31.218
	**Orientation**	0.783	.443	0.175	0.305
	**Spatial vs. Orientation**	2.549	.020	0.570	2.941
**350 ms**	**Spatial**	1.101	.285	0.246	0.396
	**Orientation**	1.348	.194	0.301	0.510
	**Spatial vs. Orientation**	0.026	.979	0.006	0.232
**500 ms**	**Spatial**	3.001	.007	0.671	6.635
	**Orientation**	4.568	< .001	1.021	143.900
	**Spatial vs. Orientation**	0.281	.782	0.063	0.241
**650 ms**	**Spatial**	3.224	.004	0.721	10.090
	**Orientation**	4.342	< .001	0.971	91.486
	**Spatial vs. Orientation**	0.227	.823	0.051	0.238

*Note.* For each cue–probe delay, the first row reports the comparison between spatial RCB index and zero, the second row reports the comparison between orientation RCB index and zero, and the third row reports the direct comparison between spatial and orientation RCB indices

### Discussion

The results of Experiment 3 showed that spatial retro-cues consistently produced robust RCBs across most cue–probe delays, including very short delays (e.g., 50 ms), replicating the pattern observed in Experiment 2. However, no significant spatial RCB was observed at the 350-ms delay. This absence likely resulted from a surprisingly high performance in the no-cue condition at the 350-ms delay, which raised the baseline used for calculating the RCB, thereby masking the cueing effect. Importantly, memory performance in the spatial retro-cue condition at the 350-ms delay was not lower than at the 200-ms delay, indicating that the lack of an RCB at this delay should not be interpreted as a performance decline under spatial cueing. Instead, the elevated baseline in the no-cue condition appears to be the key factor.

This unexpected improvement in the performance of the no-cue condition at the 350-ms cue–probe delay (950-ms memory-probe delay) was inconsistent with the patterns observed in Experiments 1 and 2, where performance in the no-cue condition remained mostly unaffected by memory–probe delay. Thus, we interpret the high performance at the 350-ms cue–probe delay in the no-cue condition as a random fluctuation rather than a systematic effect. To determine whether memory performance in the no-cue condition is genuinely modulated by cue–probe delay, a new experiment is needed to replicate and further examine this pattern.

For the orientation retro-cue condition, significant RCBs emerged only at 500 ms and 650 ms, suggesting that this type of cue requires more time to exert a benefit. These results support our hypothesis from Experiment 2: Although no orientation RCBs were observed in Experiment 2, this may have been due to the fact that participants did not encode or maintain the nonprobed feature (orientation) in VWM. In Experiment 3, the task design required participants to maintain both the probed (color) and nonprobed (orientation) features in VWM to perform the task correctly. As a result, participants showed significant orientation RCBs even when performing the same color report task as in Experiment 2.

More importantly, the results demonstrate that orientation retro-cues required a substantially longer cue–probe delay (≥500 ms) to yield reliable RCBs, in contrast to the more rapid effect of spatial retro-cues. Notably, in the offset analysis, participants’ memory performance in the orientation retro-cue condition began to improve between 200-ms and 350-ms delay, suggesting that orientation retro-cues may start to facilitate memory as early as 350 ms. However, due to the elevated baseline performance in the no-cue condition at 350 ms, no significant RCB was observed at that delay. Taken together, the findings suggest that participants require approximately 350–500 ms to use orientation retro-cues effectively and generate measurable RCBs.

Although we used identical memory arrays across Experiments 1, 2, and 3, participants’ encoding strategies likely differed across experiments. In Experiments 1 and 2, the non-probed feature (e.g., color for Experiment 1; orientation for Experiment 2) was not required for task completion—it served only to allow potential gains from feature retro-cues. Participants could complete the task even if they ignored the non-probed feature, which might have led some participants to strategically avoid encoding or maintaining it, especially in Experiment 2.

By contrast, in Experiment 3, successful task performance depended on encoding and maintaining both features, ensuring that all participants consolidated both color and orientation information into VWM. Interestingly, although we observed significant color RCBs in Experiment 1, suggesting that participants spontaneously stored color as a nonprobed feature, the lack of a requirement to do so might have influenced the observed time course of the RCB.

To address this limitation, we designed Experiment 4 as a follow-up to Experiment 1. Like Experiment 1, Experiment 4 compares the temporal dynamics of color and spatial retro-cue benefits. However, following the approach of Experiment 3, we enforced dual-feature encoding by requiring participants to encode and maintain both color and orientation features during the maintenance phase.

In addition, Experiment 4 allows us to reexamine whether, when both color and orientation features are actively maintained, performance in the no-cue condition still remains stable across memory–probe delays, or whether delay-dependent fluctuations—such as the one observed at 350 ms in Experiment 3—begin to emerge.

## Experiment 4

In Experiment 4, we revisited the temporal dynamics of RCBs for color and spatial cues under conditions that required participants to encode and maintain both color and orientation features. Compared with Experiment 1, the overall task structure remained similar: Participants were instructed to memorize four dual-feature items (color and orientation) and report the orientation of one probed item during the probe phase. However, a key modification was made to the probe display. In Experiment 1, the to-be-reported item was indicated by a white square marker appearing at the location of one memory item. In Experiment 4, this location cue in the probe display was removed. Instead, a color cue drawn from one of the items in the memory array was presented on the central orientation response bar, which participants adjusted to match the remembered orientation. This design change ensured that participants could no longer rely solely on spatial information to identify the target and were instead required to encode and maintain both the color and orientation of all items, as in Experiment 3. By enforcing dual-feature maintaining, Experiment 4 allowed us to more precisely assess the temporal dynamics of attention guidance and memory enhancement via color retro-cues—while maintaining the same memory demands as those in Experiment 3.

### Method

#### Participants

Twenty participants (ages 18–22 Years, mean age = 19.500 ± 0.224 Years; 17 women, three men) took part in Experiment 4. All participants were undergraduate or postgraduate students with normal or corrected-to-normal vision and no history of neurological or psychiatric disorders.

#### Stimuli and materials

Stimuli and materials were identical to those used in Experiment 1, except for the probe display. In this experiment, the probe consisted of a centrally presented orientation bar that participants could rotate using the mouse. The bar was rendered in the color of one of the memory items, thereby serving as a color cue to indicate the target item whose orientation was to be reported.

#### Procedure

**Fig. 7:**
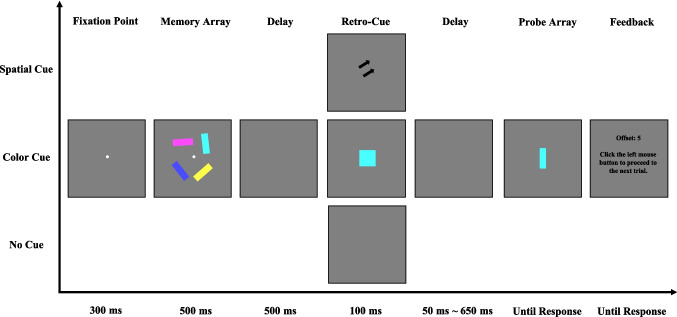
Illustration of the trial structure in Experiment 4. In the spatial cue condition, the retro-cue consisted of two arrows pointing to the location of the probed item; In the color cue condition, the retro-cue was a square filled with the color of the cued item. In the no-cue condition, no retro-cue was presented. (Color figure online)

The procedure was mostly the same as in Experiment 1 (see Fig. 7). Each trial began with a 300-ms fixation cross at the center of the screen, followed by a 500-ms presentation of the memory array. Participants were instructed to memorize both the spatial location and the visual features (color and orientation) of each item. After a 500-ms retention interval, a retro-cue (spatial or color) or no-cue was presented for 100 ms. In the spatial retro-cue condition, a white square appeared at the location of one memory item. In the color retro-cue condition, a colored square (matching one item’s color) was shown at fixation. All retro-cues were 100% valid. In the no-cue condition, the screen remained blank for 100 ms. Following a variable cue–probe delay (50 ms, 200 ms, 350 ms, 500 ms, or 650 ms), the probe display appeared. Participants were required to report the orientation of the item whose color matched the centrally presented response bar. They rotated the bar using the mouse to match the remembered orientation and confirmed their response by clicking the left mouse button. Immediate feedback was then provided, displaying the absolute angular error between the reported and correct orientation, with smaller errors indicating better memory performance. The experiment comprised 420 experimental trials, with each participant completing 28 trials in each of the 15 conditions (3 cue types: spatial retro-cue vs. color retro-cue vs. no cue × 5 cue–probe delays: 50 ms vs. 200 ms vs. 350 ms vs. 500 ms vs. 650 ms). Participants completed 20 practice trials before the main experiment to familiarize themselves with the task.

#### Data analysis

Participants’ recall performance was quantified by computing the absolute deviation between their reported orientation and the actual target orientation, referred to as “offsets,” which served as a measure of memory performance. To examine the interaction between cue type and cue–probe delay, a 3 (cue type: spatial cue, color cue, no cue) × 5 (cue–probe delay: 50 ms, 200 ms, 350 ms, 500 ms, 650 ms) repeated-measures ANOVA was conducted. Then, planned paired-samples *t* tests comparing offset values between adjacent delay intervals within each cue condition were conducted. We also compared the RCB index for each cue condition at each cue–probe delay with *t* tests, as done in Experiment 1.

### Results

**Fig. 8:**
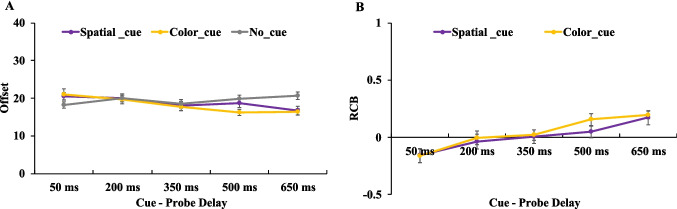
Illustration of the result of Experiment 4. Offset (**A**) and RCB index (**B**) results across different cue–probe delays for different cue type conditions. Error bars represent the standard errors of the mean. (Color figure online)

As seen in Fig. 8, a 3 (cue type: spatial retro-cue, color retro-cue, no cue) × 5 (cue–probe delays: 50 ms, 200 ms, 350 ms, 500 ms, 650 ms) two-way ANOVA on offset values revealed no significant main effect of cue type, *F*(2,38) = 2.066, *p* =.141, η_p_^2^ = 0.098, BF_incl_ = 536.661. But there was a significant main effect of cue–probe delays, *F*(4,76) = 7.179, *p* <.001, η_p_^2^ = 0.274, BF_incl_ > 1,000, and a significant interaction between cue type and cue–probe delays, *F*(8,152) = 4.215, *p* <.001, η_p_^2^ = 0.182, BF_incl_ > 1,000.

To further investigate the time course of memory performance in each cue condition, a series of planned paired-samples t-tests were conducted comparing performance across adjacent cue–probe delays. The results are summarized in Table 7. From 50 ms to 650 ms cue–probe delay, memory performance remained stable across delays in both the color retro-cue and no-cue conditions. In contrast, for the spatial retro-cue condition, performance did not change significantly between the 50-ms and 500-ms delay, but a significant improvement was observed between 500-ms and 650-ms delays (Fig. 8A).

**Table 7:** *T* tests assessing changes in performance across cue type conditions as a function of cue–probe delays in Experiment 4

Delay	Condition	*t*(19)	*p*	*d*	BF_10_
**50 - 200 ms**	**Spatial**	0.683	.503	0.153	0.286
	**Color**	1.008	.326	0.225	0.364
	**No**	1.948	.066	0.436	1.117
**200 - 350 ms**	**Spatial**	1.817	.085	0.406	0.924
	**Color**	1.969	.064	0.440	1.151
	**No**	1.283	.215	0.287	0.475
**350 - 500 ms**	**Spatial**	0.939	.359	0.210	0.343
	**Color**	1.500	.150	0.335	0.609
	**No**	1.400	.178	0.313	0.541
**500 - 650 ms**	**Spatial**	2.634	.016	0.589	3.413
	**Color**	0.229	.822	0.051	0.238
	**No**	0.911	.374	0.204	0.336

*Note.* For each adjacent delay interval, the first row reports the comparison for the offset of the spatial retro-cue condition, the second row reports the comparison for the offset of the color retro-cue condition, and the third row reports the comparison for the offset of the no-cue condition

To assess the presence and time course of RCBs, we computed an RCB index for each cue type at each cue–probe delay and conducted one-sample *t* tests against zero. In addition, we directly compared the spatial and color RCB indices using paired-samples *t* tests (Table 8). At the 50-ms cue–probe delay, the RCB indices for both the spatial and color retro-cue conditions were significantly below zero, indicating worse performance in both spatial and color retro-cue conditions than the no-cue condition. In the color retro-cue condition, the RCB index did not significantly differ from zero at the 200-ms and 350-ms delays, but significant RCBs emerged at both the 500-ms and 650-ms delays. For the spatial retro-cue condition, no significant RCBs were observed from 200 ms to 500 ms, but a significant RCB was found at the 650-ms delay. In addition, comparisons between spatial and color RCB indices revealed no significant differences at any delay, suggesting that the two cue types produced comparable levels of benefit across all cue–probe delays.

**Table 8:** *T* tests assessing RCB indices for spatial and color retro-cues across cue–probe delays in Experiment 4

Delay	Condition	*t*(19)	*p*	*d*	BF_10_
**50 ms**	**Spatial**	2.568	.019	0.574	3.040
	**Color**	2.651	.016	0.593	3.517
	**Spatial vs. Color**	0.058	.954	0.013	0.233
**200 ms**	**Spatial**	0.587	.564	0.131	0.271
	**Color**	0.064	.949	0.014	0.233
	**Spatial vs. Color**	0.459	.651	0.103	0.256
**350 ms**	**Spatial**	0.090	.929	0.020	0.233
	**Color**	0.463	.648	0.104	0.256
	**Spatial vs. Color**	0.233	.818	0.052	0.238
**500 ms**	**Spatial**	0.913	.373	0.204	0.336
	**Color**	3.057	.006	0.684	7.361
	**Spatial vs. Color**	2.006	.059	0.449	1.218
**650 ms**	**Spatial**	2.739	.013	0.612	4.106
	**Color**	6.077	< .001	1.359	> 1000
	**Spatial vs. Color**	0.362	.721	0.081	0.247

*Note.* For each cue–probe delay, the first row reports the comparison between spatial RCB index and zero, the second row reports the comparison between color RCB index and zero, and the third row reports the direct comparison between spatial and color RCB indices.

### Discussion

First, unlike Experiments 1–3, in Experiment 4 we observed that at the 50-ms cue–probe delay, participants performed significantly worse in both spatial and color retro-cue conditions compared with the no-cue condition. This suggests that the very short delay between retro-cue offset and probe onset in Experiment 4 may have introduced additional interference that impaired VWM performance. One plausible explanation is that immediately following the retro-cue, participants began shifting their attention toward the cued item. When the probe display appeared during this attentional shift, it may have acted as a competing stimulus, interfering with ongoing attentional reallocation and thus disrupting performance. In contrast, at longer cue–probe delays, the attentional shift could be completed before the onset of the probe, allowing the retro-cue to facilitate memory performance without such interference.

Importantly, we found that color retro-cues required approximately 500 ms to produce a significant RCB, which then plateaued and remained stable up to the 650-ms delay. In contrast, performance in the orientation retro-cue condition significantly improved between 500 ms and 650 ms, with a reliable RCB emerging only at the 650-ms delay. These results suggest that when participants are required to encode and maintain both color and orientation features, the deployment of attention based on color cues yields faster retro-cue benefits than that based on spatial cues. In other words, under dual-feature maintenance, the temporal dynamics of retro-cue benefits may differ by cue type, with color retro-cues being processed more rapidly than spatial cues.

Moreover, in Experiment 4, memory performance in the no-cue condition remained stable across different cue–probe delays. We did not observe the sharp performance fluctuation at a specific delay (e.g., the 350-ms delay) that was found in Experiment 3. This pattern replicates the results of Experiments 1 and 2, suggesting that the enhanced memory performance at the 350-ms delay in the no-cue condition of Experiment 3 was likely a random fluctuation that is difficult to replicate. Overall, the results support the view that memory performance in the no-cue condition is not sensitive to small variations in the length of the memory-probe delay. This finding is consistent with the results reported by Zhang and Luck (2009), who systematically manipulated memory-probe delays in a color-and-shape report task. They found that memory performance remained stable as the delay increased from 1 to 4 s, and only began to decline when the delay extended from 4 to 10 s.

## General discussion

The primary goal of the present study was to examine the temporal dynamics of RCBs by directly comparing spatial retro-cues with two types of feature-based retro-cues—color and orientation. Our aim was to evaluate whether spatial cues differ in effectiveness and processing speed from these two types of feature cues. We reasoned that if one retro-cue type is more effective or can be used more rapidly than another, such an advantage should manifest in two ways. First, the more effective cue would elicit a significant RCB at shorter cue–probe delays than the less effective one. Second, at these shorter delays, the RCB index would be significantly greater for the more effective cue type compared with the less effective one. According to these criteria, Experiments 1 and 4 both provided evidence that color retro-cues led to more efficient and faster retro-cue benefits than spatial retro-cues. Similarly, Experiments 2 and 3 revealed that spatial retro-cues were more effective and faster than orientation retro-cues. Taken together, these findings suggest that although both color and orientation cues are categorized as feature retro-cues, the time required for them to produce RCBs—and the underlying processing mechanisms—may differ substantially. Therefore, in the following sections, we discuss the results for color and orientation retro-cues separately.

The findings of color retro-cue support our first hypothesis, which posits that feature retro-cues—particularly color—can guide attention more directly and rapidly than spatial retro-cues by bypassing spatial transformations (Carrasco, 2011). As a perceptually salient and automatically processed feature (Andersen et al., 2009), color likely engages parallel selection mechanisms that allow for fast and efficient isolation of the relevant item in memory. In contrast, spatial retro-cues typically require transforming symbolic spatial indicators (e.g., arrows) into corresponding memory locations (Wang & van Ede, 2025), a process that introduces temporal cost and delays the emergence of RCBs.

However, orientation retro-cues failed to produce benefits unless participants were explicitly required to encode both color and orientation features (as in Experiment 2), and even then, the benefits emerged more slowly compared with those from spatial retro-cues. This discrepancy underscores the limitations of treating “feature retro-cues” as a unified category. Orientation features lack the perceptual salience and automaticity of color processing. Hulleman (2020) found that orientation-based visual search is significantly slower than search based on color or motion information, suggesting that orientation is a less efficient guiding attribute. The effectiveness of feature-based retro-cues may depend on similar mechanisms—selectively activating all memory representations sharing a given feature—thus making orientation cues inherently slower and less robust in guiding attention within VWM. When participants could freely choose encoding strategies (Experiment 2), they likely prioritized color—a strategy that minimized cognitive load but rendered orientation cues ineffective. However, when forced to encode both features (Experiment 3), orientation retro-cues produced measurable RCBs at 500-ms delays or longer. In contrast to the relatively direct processing of spatial retro-cues, feature-based retro-cues may engage more complex and resource-intensive cognitive mechanisms, thus requiring longer temporal delays to produce RCBs (Heuer et al., 2016). These findings suggest that the effectiveness of feature retro-cues varies depending on the perceptual salience and complexity of the cued feature. These differences likely stem from the unique processing characteristics of each feature and their corresponding attentional guidance mechanisms. First, orientation cues lack the highly salient properties of color cues, which naturally captures participants’ attention, making color cues almost impossible for participants to ignore. Additionally, due to their stimulus-driven nature, color cues are processed highly efficiently—participants can quickly identify the cued item without needing to sequentially search through all the items maintained in VWM. Conversely, orientation processing lacks the automaticity and salience of color, making orientation cues inherently more difficult to use (Hao et al., 2018; Miller et al., 2014; Vogel et al., 2006). Effectively using orientation cues may require participants to individually compare the cued feature against all stored representations. This searching significantly extends the time required to effectively use orientation cues, even when participants are motivated to do so. Spatial retro-cues, by comparison, rely on locational information to guide attention to the cued item. Spatial cues involve a single transformation step, wherein the spatial location of the retro-cue is converted into the corresponding memory item. This intermediary process takes longer than the direct cueing of color, it is still more efficient than the cognitively demanding processes involved in using orientation cues.

It is worth noting that prior research has provided limited evidence regarding the time required for feature RCBs to emerge. For instance, Pertzov et al. (2013) reported that a cue–probe delay of 1,000 ms was necessary to observe a significant color RCB. In contrast, our results demonstrate that a reliable color RCB can emerge with a delay as short as 200 ms. This suggests that participants can access and utilize color retro-cues more rapidly than previously thought. The discrepancy with Pertzov et al. (2013)‘s findings may be attributable to their relatively small sample size (n = 12), which might have required longer delays and more stable RCBs to reach statistical significance. To our knowledge, no previous studies have systematically examined the time course required for orientation-based RCBs. Our findings indicate that orientation RCBs may require up to 500 ms of cue–probe delay to reliably emerge. This is broadly consistent with findings from Arnicane and Souza (2021), who reported significant RCBs at a 500-ms cue–probe delay. Taken together, these findings suggest that the time needed for feature-based RCBs depends on several factors, including the type of feature cue and the nature of the memory report task (e.g., whether participants are asked to report color or orientation). Nevertheless, to ensure robust and consistent feature-based RCBs, a cue–probe delay of at least 500-ms cue–probe delay appears to be a reliable benchmark.

Moreover, the markedly different onset times of RCBs between Experiment 1 and Experiment 4 provide further insights. In Experiment 1, the color RCB index was already significantly greater than the spatial RCB index at the shortest cue–probe delay of 50 ms, and both color and spatial RCBs became stable by 200-ms cue–probe delay. In contrast, in Experiment 4, the color RCB didn’t emerge until the 500-ms delay, and the spatial RCB didn’t emerge until 650-ms delay. We propose that this discrepancy stems from differences in task demands and retrieval processes across the two experiments. In Experiment 1, participants had to first identify the target item based on the location indicated by a white square in the probe display, and then recall its associated orientation. This two-step process—first localization, then recall—was relatively complex and introduced higher cognitive load in the no-cue condition. Furthermore, although the white square provided helpful spatial information, it might also have interfered with the precise retrieval of the orientation feature, thereby impairing performance in the no-cue condition. Under the retro-cue condition, participants could rely on the cue to pre-select the to-be-probed item before the probe display appeared. This advance selection likely reduced memory load (Kuo et al., 2012; Souza, Rerko, Oberauer et al., 2014b; Williams et al., 2013) or shielded the target representation from interference by the white square (Makovski & Jiang, 2008; Makovski & Pertzov, 2015), leading to the emergence of RCBs at shorter delays. By contrast, in Experiment 4, the white square was removed, and participants could directly retrieve the orientation based on the color without resolving any spatial information. This more direct retrieval approach simplified the task and significantly reduced cognitive demands, allowing participants to maintain high memory accuracy even in the absence of a retro-cue. This is evidenced by the significant lower mean offset values in Experiment 4 (19.46 ± 3.42) compared with Experiment 1 (31.53 ± 8.46), *t*(36) = 5.882, *p* <.001, Cohen’s *d* = 1.911, BF_10_ > 1,000, suggesting better overall performance in the no-cue condition and thus a higher baseline.

Based on this, we suggest that in Experiment 4, due to the overall simplicity of the task, participants could already perform well at very short cue–probe delays (e.g., 50 ms), at which point the retro-cue might not yet have been fully processed or integrated into memory updating—potentially making it ineffective or even distracting. As the cue–probe delay increased, the cue became more fully used. However, at intermediate delays (e.g., 200–350 ms), memory performance in the no-cue condition remained near ceiling, leaving little room for improvement, and thus RCBs were not yet evident. Only at longer delays (e.g., 500 ms), when memory performance in the no-cue condition began to decline, did the retro-cue demonstrate its benefit by preventing further forgetting. In contrast, in Experiment 1 both spatial and color retro-cues showed clear and stable effects by 200 ms. This earlier emergence of RCBs can be attributed to the higher task demands in Experiment 1, particularly due to the interference caused by the white square in the no-cue condition during feature retrieval. However, in the retro-cue conditions, participants had already prioritized the cued item during the cue–probe delay, allowing them to resist the interference of the white square in the probe display and thereby benefit earlier from the cue.

Notably, a similar task manipulation was applied in Experiments 2 and 3, though task demands and retrieval processes differed. One might expect Experiment 3 (with a simplified retrieval process) to yield better performance than Experiment 2. However, the opposite pattern was observed: mean offset values in the no-cue condition were significantly higher in Experiment 3 (61.95 ± 12.56) than in Experiment 2 (47.17 ± 8.07), *t*(39) = 4.505, *p* <.001, Cohen’s *d* = 1.408, BF_10_ = 342.3. This apparent difference may be explained by participants’ encoding strategies. Gao et al. (2011) proposed that VWM is not a passive store of perceptual input but plays an active role in online perception. That is, participants can actively adopt strategies during encoding to improve memory performance or reduce task load. Given that our task provided trial-by-trial feedback on recall error, participants in Experiment 2 may have realized that encoding both features increased their overall error. As a result, they were likely motivated to reduce cognitive load by prioritizing the easier and task-relevant feature—color—thus improving recall performance. This interpretation is supported by Marshall and Bays (2013), who showed that encoding multiple features simultaneously can impair the recall of each due to increased memory load.

Taken together, these findings suggest that participants in Experiment 2 may have intentionally avoided binding orientation and color features in order to optimize memory for the target feature and minimize cognitive effort. In contrast, participants in Experiment 3 were explicitly required to encode both color and orientation, which increased memory load despite the simpler retrieval structure. This likely explains the overall decline in performance compared to Experiment 2. In Experiment 1, where orientation was the probed feature, participants may have been less successful in ignoring the nonprobed color feature. Although they may have attempted a similar strategy of selective encoding, color—due to its salience and surface-level nature—was likely encoded automatically and was harder to suppress, even when irrelevant to the task. As such, participants in Experiment 1 may have lacked both the motivation and the ability to disregard color. It is important to emphasize that direct comparisons across the four experiments should be made with caution, as differences in task structure and participant samples limit the interpretability of cross-experiment effects. Rather than focusing on between-experiment contrasts, our primary aim was to examine the relative effectiveness and temporal dynamics of spatial and feature-based retro-cues within each individual experiment.

We also observed that in some conditions, retro-cues presented with a short cue–probe delay of only 50 ms could even interfere with performance. This may be due to the limited cue presentation duration of 100 ms, which might be insufficient for some retro-cues successfully encoding. After the retro-cue disappeared, participants may have needed to use part of the cue–probe delay period to consolidate the cue representation through visual afterimages before retrieving the associated memory item. Alternatively, cue consolidation and memory retrieval might have occurred simultaneously. If the cue–probe delay period is too short, participants may not have completed the retrieval process by the time the probe appears, leading to a failure in effectively utilizing the retro-cue. Based on this reasoning, we propose that differences in the time course of RCBs across cue types may partially reflect differences in cue encoding speed. The faster the cue is encoded, the less of the delay it occupies, allowing RCBs to emerge earlier.

Although we did not find direct empirical evidence showing that color cues are encoded more quickly than orientation cues, color is widely regarded as a highly salient feature that is typically processed rapidly. In contrast, orientation is less salient and may require more time to be integrated and stabilized in memory. This assumption is indirectly supported by previous studies using retro-cues, which typically present color or spatial cues for 100 ms, suggesting that 100 ms is sufficient for effective encoding of color and spatial cues (Pertzov et al., 2013; Souza, Rerko, & Oberauer et al., 2014b; van Moorselaar et al., 2015). However, this duration may not be adequate for encoding orientation cues. Liu et al. (2007) in a precueing study, found that spatial cues significantly improved reaction time and accuracy with cue–target delays of 300 ms, whereas motion directional cues required at least 500 ms to produce significant behavioral benefits. These results indicate that spatial attention can be deployed rapidly, whereas attention based on motion direction may require a longer time to integrate relevant information or activate the appropriate feature template in working memory. This pattern generally aligns with our results: In Experiment 3, spatial cues produced significant RCBs in the 50-ms cue–probe delay condition, whereas orientation cues required cue–probe 500 ms. Therefore, we suggest that orientation cues require more time to be effectively encoded than color or spatial cues, which may explain the delayed onset of RCBs for orientation.

This leads to a further question: If sufficient encoding time is provided, could orientation cues lead to faster RCBs than spatial cues? While our study was not specifically designed to address this question, the results of Experiment 3 offer a basis for speculation. In that experiment, spatial cues produced RCBs at 50 ms, while orientation cues required 500 ms—a difference of 450 ms. Even assuming that orientation cue encoding takes up to 300 ms, there would still be an additional 150–200 ms unaccounted for, suggesting that orientation cues may involve additional cognitive demands beyond encoding alone. Thus, even when given sufficient encoding time, orientation cues still do not reach the speed of spatial cues in producing RCBs. In sum, our findings suggest that differences in cue encoding speed across feature types can significantly influence the timing of RCBs, while this does not alter our core conclusion—that color cues produce earlier RCBs than spatial cues, and spatial cues earlier than orientation cues.

Another noteworthy consideration in our design is the type of information provided in the probe display across experiments. In Experiments 1 and 2, the probe display included a white square that served as an exogenous spatial cue, indicating the location of the item to be reported. In contrast, in Experiments 3 and 4, the probe display included a feature cue (an orientation cue in Experiment 3 and a color cue in Experiment 4) to indicate the target item. This raises a natural question: Does the type of remembered information (spatial vs. feature) modulate the mechanisms of RCB? Specifically, when the retro-cue type matches the type of cue presented in the probe display, does this consistency provide an additional performance advantage? According to this hypothesis, one would expect spatial retro-cues to facilitate faster memory access when participants ultimately rely on spatial information to identify the target item, as in Experiments 1 and 2. Conversely, feature retro-cues might offer greater advantages in tasks where the probe relies on feature information, as in Experiments 3 and 4. To evaluate this idea, we compared retro-cue performance across matched pairs of experiments—namely, Experiments 1 and 4 (where the report target was orientation information), and Experiments 2 and 3 (where the report target was color information). Behaviorally, for spatial retro-cues, participants in Experiment 1 showed significantly worse performance (offset = 23.99 ± 5.87) than those in Experiment 4 (offset = 18.78 ± 4.16), *t*(36) = 3.180, *p* =.003, Cohen’s *d* = 1.033, BF_10_ = 12.72. Similarly, spatial retro-cue performance in Experiment 2 (offset = 27.09 ± 8.09) was significantly better than in Experiment 3 (offset = 48.18 ± 16.45), *t*(39) = 5.249, *p* <.001, Cohen’s *d* = 1.640, BF_10_ > 1,000. These results suggest that performance in the spatial retro-cue condition was better when the probe provided feature-based cues (as in Experiment 4) than when it provided spatial cues (as in Experiment 1), at least when participants reported orientation. The opposite pattern was observed when participants reported color: performance in the spatial retro-cue condition was better when the probe provided spatial cues (Experiment 2) than when it provided feature cues (Experiment 3). For feature retro-cues, a similar pattern emerged only in part. The color retro-cue in Experiment 1 yielded a significantly larger offset (24.53 ± 6.86) compared with that in Experiment 4 (18.20 ± 3.76), *t*(36) = 3.574, *p* =.001, Cohen’s *d* = 1.161, BF_10_ = 30.54, suggesting that performance in the color retro-cue condition was better when the probe provided a matching feature cue. However, no significant difference was observed between the orientation retro-cue conditions in Experiment 2 (48.97 ± 10.62) and Experiment 3 (43.35 ± 15.24), *t*(39) = 1.071, *p* =.291, Cohen’s *d* = 0.335, BF_10_ = 0.482, indicating that the consistency between retro-cue and probe cue had limited influence when the reported feature was orientation. Taken together, these results suggest a more nuanced picture. The potential advantage of spatial retro-cues when spatial information is used at test appears to be influenced by the type of to-be-reported feature. However, feature retro-cues—particularly color—may have an advantage when the probe cue matches the retro-cue type, but this effect is not robust across all feature types (e.g., orientation).

It is important to note, however, that these comparisons across experiments were conducted between participants and across different experimental tasks. Thus, any observed effects may reflect not only retro-cue and probe cue consistency but also individual differences and task-specific factors. As such, we refrain from drawing strong conclusions regarding cue-type consistency effects on retro-cue benefits. Future studies that systematically manipulate the consistency between retro-cues and probe cues within subjects will be necessary to clarify whether and how such consistency influences the underlying mechanisms and temporal dynamics of RCB.

Another important question concerns the theoretical implications of our findings for existing hypotheses regarding the mechanisms underlying RCBs. Several accounts have been proposed to explain RCBs, including active maintenance (Makovski & Jiang, 2008; Rerko et al., 2014; Souza, Rerko, Oberauer et al., 2014b), removal of irrelevant items (Fu et al., 2022; Kuo et al., 2012; Williams et al., 2013), and interference reduction (Makovski & Jiang, 2008; Makovski & Pertzov, 2015). Our experiments contribute to this literature by demonstrating that RCBs—whether induced by spatial or feature retro-cues—do not occur instantaneously. Instead, they appear to reflect a dynamic, time-dependent process involving attentional modulation and memory reorganization. Across our experiments, we consistently observed that RCBs emerged and increased with longer cue–probe delays, before reaching a plateau. For instance, color retro-cue benefits increased from 50 ms to 200 ms and then stabilized, while spatial retro-cue benefits plateaued after 200 ms. In contrast, memory performance in the no-cue condition remained stable across delays, suggesting that retro-cues may help prevent memory decay. This pattern is consistent with the attentional refreshing hypothesis (Matsukura et al., 2007; Pertzov et al., 2013; Rerko & Oberauer, 2013), which proposes that retro-cues elicit iterative attentional boosts to sustain vulnerable memory representations and counteract temporal degradation.

It is also worth highlighting that in Experiment 3, participants were required to encode and maintain both color and orientation features, whereas in Experiment 2, only color was task-relevant. This increased cognitive demand may have necessitated additional operations, such as feature binding. This raises a critical theoretical question: Do the mechanisms underlying RCBs differ between feature-binding tasks (e.g., color–orientation conjunctions) and single-feature tasks (e.g., color only)? Binding tasks are likely to engage supplementary cognitive processes, including neural reentry mechanisms that support the integration of multiple features into coherent object representations. Notably, in Experiment 2, if participants ignored the orientation information, the task closely resembles a conventional single-feature paradigm using color stimuli with spatial retro-cues. We argue that the mechanisms supporting RCBs in feature-binding tasks may diverge from those in single-feature tasks. In feature-binding contexts, the successful use of a retro-cue requires not only prioritizing the target item but also maintaining correct feature conjunctions. Specifically, attention must be directed to a target object based on one feature dimension (e.g., color), and the corresponding feature (e.g., orientation) must be retrieved accurately. A failure to maintain the integrity of feature bindings—despite accurate storage of individual features—would preclude effective use of the retro-cue and consequently diminish the likelihood of observing an RCB. In contrast, such dependency on binding integrity is absent in single-feature tasks. Moreover, most previous investigations into the mechanisms of RCBs have been conducted using spatial retro-cues in single-feature tasks (Souza & Oberauer, 2016). Our findings indicate that the temporal dynamics—and potentially the underlying cognitive mechanisms—of RCBs differ depending on the type of cue employed. Specifically, we observed distinct time courses and cueing efficiencies for spatial versus feature-based retro-cues. These differences call into question the generalizability of conclusions drawn solely from spatial retro-cue paradigms to other cueing contexts. We therefore encourage future studies to expand the scope of RCB research by systematically examining different types of feature-based cues. This broader approach will be crucial for evaluating whether and how the mechanisms underlying RCBs vary between feature-binding and single-feature memory tasks.

## Conclusions

The present study systematically examined the temporal dynamics of RCBs in VWM by comparing spatial cues with two types of feature retro-cues: color and orientation. Across four experiments, we found that different cue types varied in both the speed and magnitude of their effects. Importantly, our results suggest that a cue–probe delay of at least 500 ms may be necessary to reliably observe feature RCBs, particularly for orientation retro-cues. Color retro-cues produced the fastest and most robust RCBs, followed by spatial cues, with orientation cues yielding the slowest and most limited benefits. These results demonstrate that not all feature retro-cues function equivalently and challenge the notion of a unified feature cue category. The onset timing of RCBs was not solely determined by cue type but was also shaped by task demands and retrieval processes. In particular, the same retro-cue type produced markedly different benefits depending on whether the target feature was color or orientation, and whether the probe display provided spatial or feature cues. Taken together, our findings advance current understanding of the mechanisms underlying attentional prioritization in VWM, which suggest that the temporal efficiency of retro-cueing is jointly determined by cue type, retrieval format, and the cognitive demands imposed by the task. These results underscore the importance of considering the distinct cognitive operations associated with different cue types when evaluating models of selective attention in working memory.

## Data Availability

Anonymized data are available on the OSF (https://osf.io/rwmkx).

## Code availability

The experimental code are available on the OSF (https://osf.io/rwmkx).
